# Evaluation of a novel very high sun-protection-factor moisturizer in adults with rosacea-prone sensitive skin

**DOI:** 10.2147/CCID.S134857

**Published:** 2017-06-10

**Authors:** Mathieu Grivet-Seyve, Francine Santoro, Nadège Lachmann

**Affiliations:** 1 Galderma Research and Development, Sophia Antipolis, France; 2 Galderma Research and Development, Egerkingen, Switzerland

**Keywords:** rosacea, photodamage, UV light, sensitive skin, patient-reported outcomes, moisturizer

## Abstract

**Background/objective:**

Rosacea-prone sensitive skin requires high sun-protection factor (SPF) moisturizers. This study evaluated Daylong Extreme SPF 50+ lotion, a novel cream containing five ultraviolet filters, two emollients, and three skin conditioners.

**Subjects and methods:**

This was an open-label, single-center study. On day 1, before treatment, subjects answered a questionnaire on their skin conditions and sunscreen habits, and both subjects and dermatologist evaluated skin status. Subjects applied the product once daily in the morning to the face for 21 days, and after approximately 3–5 minutes they assessed tolerability and short-term cosmetic acceptability in a questionnaire and daily diary. On day 22, the dermatologist and subjects evaluated skin status for long-term tolerance and cosmetic acceptability.

**Results:**

The study enrolled 44 individuals (mean age 58.8 years, 91% female). At baseline, most subjects (39 of 44) showed erythema, and ~30% showed dryness and scaling. Dermatologists noted four cases of pustules and one case of papules. After 21 days’ treatment with the product, the dermatologist reported significantly less erythema, dryness and scaling, three cases of pustules and two cases of papules. At baseline, ~75% of subjects noted a feeling of dryness, >50% reported tension, and nearly 25% reported tickling. After using the product for 21 days, subjects reported significantly less tension, dryness, and tickling. Some subjects noted itching and burning before and after using the product. One subject noted papules during treatment. Most subjects said that the product was pleasant, did not irritate the skin or cause stinging/burning, was easy to apply, quickly absorbed, and nongreasy, improved skin moisturization, helped prevent sun-provoked facial redness, did not worsen rosacea, and was easily incorporated into their skincare regimen. Half would switch to the product, and 80% of subjects would buy and recommend the product.

**Conclusion:**

The product was well tolerated in rosacea-prone subjects, producing objective and subjective improvements in skin status and symptoms.

## Introduction

Approximately 5%–10% of the population suffers from rosacea, a chronic inflammatory skin disorder.^1^,^2^ Rosacea is most prevalent among people aged 30–50 years, with fair skin, and of northern European ethnic origin. However, rosacea can also emerge at any age and in people of Asian and African ethnic backgrounds.^3^,^4^ For example, Kyriakis et al reported that the median age of 50,237 outpatients with rosacea was 59 years in males, with an age range of 14–95 years, and 48 years for females, with an age range of 8–89 years.^5^ Traditionally, women are said to be approximately two to three times more likely to develop rosacea than men.^3^ However, Kyriakis et al found that the sexes were equally likely to develop rosacea, although women were more likely than men to present with rosacea between 36 and 50 years of age.^5^

The chronic inflammation that underlies rosacea can result in a variety of signs and symptoms, including flushing, telangiectasia, inflammatory lesions, and ocular manifestations.^6^ Many signs and symptoms of rosacea are also associated with other dermatological conditions. However, two features seem to be diagnostic of rosacea: persistent erythema affecting the central face that shows periodic intensification, and phymatous rosacea (excess tissue), especially affecting the nose.^6^

As rosacea affects facial appearance, the condition can have a marked psychological impact and undermine health-related quality of life.^3^,^7^–^9^ As Moustafa et al commented, “… patients with dermatologic conditions bear their disease for the world to see”.^7^ In a meta-analysis encompassing 1,716 patients, 43% had at least moderately impaired health-related quality of life.^8^ In addition, rosacea patients can experience low self-esteem, embarrassment, depression, stress, or anxiety.^3^,^7^,^8^ Some patients may avoid social situations because of rosacea or develop social phobia.^3^,^7^ In the meta-analysis, 62% of people with severe rosacea (based on patient self-assessment) reported that erythema at least somewhat interfered with their social life. Furthermore, 48% of people with severe rosacea reported that erythema at least somewhat interfered with their work life.^8^ Despite this potentially profound impact, an estimated 82% of rosacea patients are untreated.^1^

Rosacea management encompasses patient education and skincare (eg, using moisturizers and sunscreen), and may also involve drugs, surgery, or both.^2^ It has been recently recognized by the Rosacea Consensus international panel of experts that education and instruction of all rosacea patients in proper skincare use is essential for enhancing therapeutic outcomes. The elements of essential skincare are: 1) known trigger avoidance, 2) use of sunscreen with at least sun-protection factor (SPF) 30, 3) frequent use of moisturizers, and 4) use of gentle over-the-counter cleansers. Dermatologists advise patients who frequently flush or have a family history of rosacea to identify and avoid physiological and environmental triggers.^2^ For instance, rosacea patients commonly report heightened skin sensitivity to many common skincare and personal hygiene products,^10^ such as products containing alcohol, menthol, peppermint, eucalyptus, and clove oils.^2^ Indeed, 24%–41% of patients suggest that skin products exacerbate their rosacea.^3^,^11^ Therefore, skin products should not contain ingredients likely to induce irritant or allergic contact dermatitis, and should be cosmetically pleasing.^10^

In addition, patients with rosacea often experience dry facial skin, which can exacerbate symptoms.^12^ Moisturizers can repair and maintain stratum corneum-barrier function, enhance skin hydration, and reduce the likelihood of skin irritation, such as that associated with topical medications.^10^ As a result, moisturizers relieve dry skin and improve dermatological characteristics, such as softness and suppleness.^12^ Increasing evidence also suggests that moisturizers may improve skin homeostasis and repair defective barrier function, and can be adjuvants to other therapies in the management of rosacea.^12^

Skincare products for people with rosacea should also block ultraviolet (UV) light. Sun exposure is the leading flare trigger, cited as a contributory factor by 61%–81% of patients.^3^,^11^ For instance, levels of cathelicidin LL37 – a peptide with a broad spectrum of antimicrobial action that is involved in innate immunological responses – are markedly increased in skin affected by rosacea compared with unaffected controls.^13^ Cathelicidin LL37 modulates the proinflammatory and proangiogenic effects of UV radiation and thereby contributes to enhanced sensitivity to sun exposure in rosacea. Application of sunscreens reduces LL37 production and in turn reduce production of the reactive oxygen species that can trigger rosacea.^2^ Therefore, rosacea patients should use photoprotection, which includes applying broad-spectrum sunscreens with a minimum SPF of 30.^11^ Against this background, this open-label, single-center investigation assessed 3 weeks’ treatment with a novel SPF 50+ moisturizer (Daylong Extreme SPF 50+ lotion) in adults with rosacea-prone sensitive skin.

## Subjects and methods

The study was performed at the ProDerm Institute for Applied Dermatological Research GmbH, Schenefeld/Hamburg, Germany between June 11 and July 7, 2015. Galderma Research and Development funded the study. No individual ethics committee review was necessary, as the study was investigating a cosmetic product, as defined by European cosmetic regulation EC 1223-2009. This study was conducted in accordance with the ethical principles derived from the Declaration of Helsinki and International Council for Harmonisation of Technical Requirements for Pharmaceuticals for Human Use good clinical practices and in compliance with local regulation. All subjects provided their written informed consent before entering the study.

### Product and application

Daylong Extreme SPF 50+ lotion is a water-based cream containing five UV filters, two emollients, and three ingredients that condition the skin. Subjects applied the product once daily in the morning to their face, excluding the area around the eyes, at home for 21 days. The last application was on the day before the last scheduled visit (day 22). Materials were weighed at the start and end of the study, and the amount of product used was estimated.

Subjects were able to continue to use their normal cleansers (eg, soaps, shampoos, bath and shower products), leave-on cosmetics (eg, creams, lotions, oily cleansing products), and decorative cosmetics (eg, mascara, makeup, cover cream, tinted day cream). However, subjects were asked to leave an interval of approximately 5 minutes before or after applying the product and not to change their brand or use new products during the study. Participants were also asked to avoid excessive sun exposure, therapy with UV light, and artificial tanning.

### Screening

Exclusion criteria included acne, seborrheic dermatitis, and diseases that might require regular medication, such as lupus, psoriasis, and atopic dermatitis. Subjects were excluded if they had received medical treatment for rosacea within the 6 months preceding the study or if they had used any topical medication at the test area or received systemic therapy with immunosuppressive drugs (eg, corticosteroids), antihistamines, or antibiotics within the 4 weeks before the start of the study. Subjects were also excluded if they had used anti-inflammatories or analgesics (except for minor pain-relief medicine, such as aspirin or paracetamol) within the 3 days before the start of the study.

All subjects included exhibited Fitzpatrick I–III skin that was rosacea-prone; in other words, the subjects reported flushing easily. In addition, subjects reported that at least one of the following triggered flushing: sun exposure, emotional stress, or hot weather. They also reported being sensitive to cosmetic products applied on the face. The Supplementary material includes the screening questionnaire (Table S1).

Subjects underwent a stinging test to confirm sensitive skin. The dermatologist applied lactic acid (10%) to one nasolabial fold and infraorbital cheek using a cotton-tipped swab. The dermatologist then applied physiological saline solution to the contralateral side. The swab was dipped into the lactic acid or physiological saline solution and then applied to the upper end of the nasolabial fold, about 3 cm under the inner corner of the eye, and moved in a rotating motion across the infraorbital part of the cheek to about 3 cm under the outer corner of the eye and back. The swab was then moved along the fold of the nostrils and back, and along the nasolabial fold to the side of the corner of the mouth and back, ending at the point where application started. The movement was repeated once. The subject reported any sensation at 10 seconds and 5 and 10 minutes after application. Subjects who reported stinging with lactic acid but not saline at any of these times were defined as having sensitive skin.

### Study visits

Before treatment began on day 1, the dermatologist evaluated each of the following dermatological signs on a 5-point scale (0, no reaction; 0.5, very slight; 1, slight; 2, moderate; 3, strong): erythema, dryness, scaling, fissures, papules, pustules, edema, vesicles, weeping, and “other” aspects of skin status. Subjects answered a questionnaire about sunscreen habits (see Tables S1–S6) and evaluated each of the following on a 5-point scale (0, no reaction; 0.5, very slight; 1, slight; 2, moderate; 3, strong): itching, burning, tension, tickling, feeling of dryness, other. Subjects then applied the product. Approximately 3–5 minutes later, subjects assessed local tolerability and cosmetic acceptability using a questionnaire (see Table S5).

On day 22, the same dermatologist that performed the initial evaluation and subjects evaluated skin status on the 5-point scale. Subjects also assessed local tolerability and cosmetic acceptability using a questionnaire (see Table S6). Timings could deviate by ±2 days. Subjects documented the daily application of the product and any reactions and signs of discomfort in a diary.

### Statistics

Statistical analyses were performed using SAS for Windows software. Wilcoxon signed-rank tests were used to compare results at each time point. *P*<0.05 was considered to indicate a statistically significant difference. Binomial tests compared frequencies of results on the questionnaires.

## Results

### Demographics and baseline characteristics

The study enrolled 44 subjects, all of whom completed the study and were included in the analysis. One subject stopped using the formulation, but attended the follow-up appointment. The subjects’ mean age was 58.8±9.7 years. Forty of the subjects (91%) were female.

At baseline, all subjects agreed that their facial skin was sensitive to topical cosmetic products and that they preferred to use well-tolerated products on their face. All but one subject (98%) agreed that they flushed strongly or got a red face in the sun. Significantly more subjects agreed than disagreed with these statements (all *P*<0.001; see Tables S4–S6). On average, participants used 50 g of the product during the study.

### Dermatologist assessment

**Figure 1:**
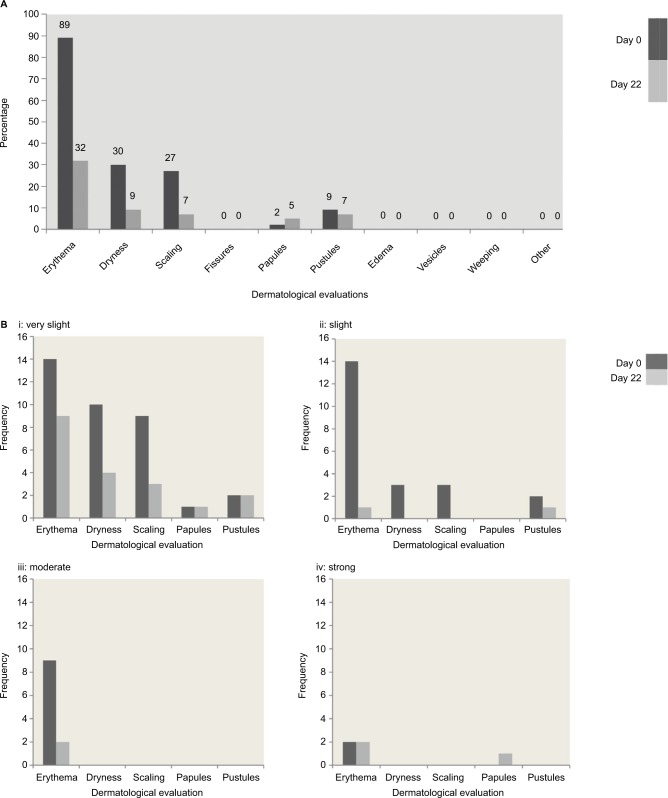
Dermatological evaluation of skin status at baseline (day 0) and day 22. **Notes:** (**A**) Dermatological evaluation of reactions on day 1 and day 22. (**B**) Dermatological evaluation of skin status, with frequency of: i= very slight, ii= slight, iii= moderate, and iv= strong skin reactions. Evaluation was based on a 5-point scale: 0, no reaction; 0.5, very slight; 1, slight; 2, moderate; 3, strong. There were no cases reported of fissures, edema, vesicles, weeping, or “other”.

At baseline, most participants showed erythema, and around 30% showed dryness and scaling. Dermatologists noted four cases of pustules and one case of papules (see Table 1 and Figure 1). Participants did not show fissures, edema, vesicles, or weeping. On day 22, the dermatologist identified significantly less erythema, dryness, and scaling after subjects had used the product for 21 days compared with baseline. No further significant differences were found. There were three cases of pustules and two cases of papules. Most subjects had no observable reaction on day 22, and for those who had reactions, the severity was mostly “very slight” or “slight” (Table 1, Figure 1).

### Subject assessment

**Figure 2:**
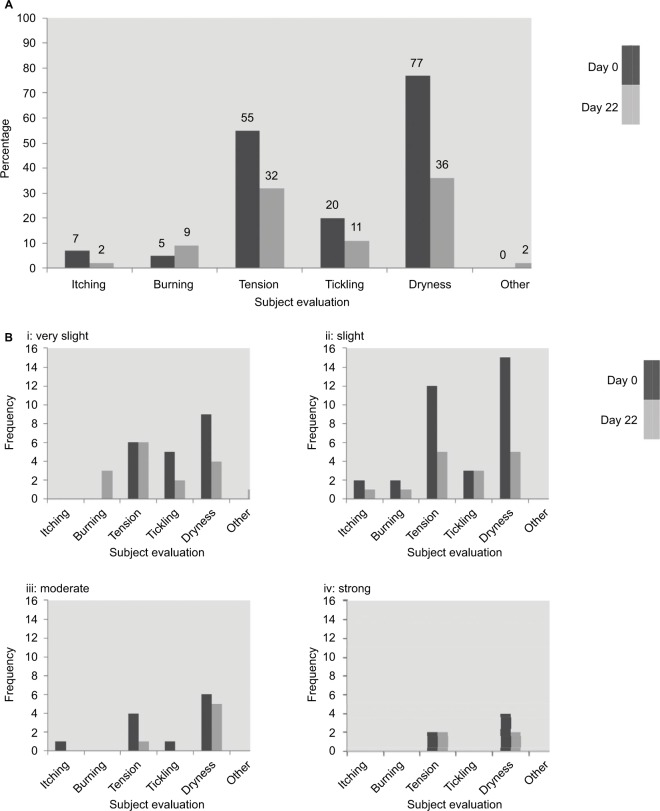
Subject evaluation of skin status at baseline (day 0) and day 22. **Notes:** (**A**) Subject evaluation of reactions on day 1 and day 22. (**B**) Subject evaluation of skin status, with frequency of: i= very slight, ii= slight, iii= moderate, and iv= strong skin reactions. Evaluation was based on a 5-point scale: 0, no reaction; 0.5, very slight; 1, slight; 2, moderate; 3, strong.

At baseline, more than three-quarters of subjects noted a feeling of dryness in the test area, more than half reported tension, and nearly a quarter reported tickling (Table 2, Figure 2). Subjects reported significantly less tension, dryness, and tickling after using Daylong Extreme SPF 50+ lotion once daily for 21 days compared with baseline. No other significant differences were found. Some participants noted itching and burning before and after using the product. One subject noted papules during treatment (Table 1). Subjects assessed the severity of most outcomes as “very slight” or “slight”. Most of the subjects reported no reaction on day 22, and for those who had reactions the severity was mostly “very slight” or “slight” (Table 2, Figure 2).

### Cosmetic acceptability

The Supplementary material contains detailed results for the cosmetic acceptability questionnaires. Significantly more subjects had a pleasant than an unpleasant feeling (including texture, color, and scent) of the product following their first application and after using the product for 21 days. Significantly more subjects agreed than disagreed that the product did not irritate the skin, did not cause stinging or burning, was easy to apply, was quickly absorbed, and did not make the skin appear greasy.

**Figure 3:**
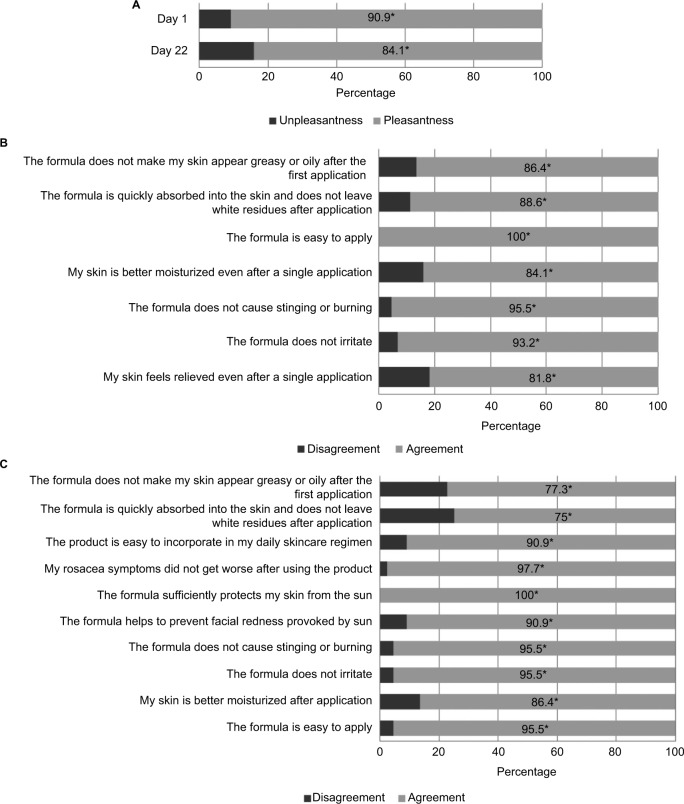
Subjects’ assessment of cosmetic acceptability of Daylong Extreme SPF 50+ lotion after a single application and once-daily treatment for 21 days. **Notes:** *P<0.001. (**A**) Overall impression on days 1 and 22. (**B**) Question responses on day 1. (**C**) Question responses on day 22.

Subjects reported that the product improved skin moisturization, helped prevent facial redness provoked by the sun, did not worsen rosacea symptoms, and was easily incorporated into their daily skincare regimen (Figure 3). In addition, 80% of participants would buy the product and recommend it to family and friends. Half would switch to the product.

### Tolerability

Daylong Extreme SPF 50+ lotion was well tolerated. One subject discontinued the product after developing pustules on the chin and papules on the left cheek on day 16. The reaction was considered to be possibly related to the product. However, it was considered that due to the subject’s skin status, they should not have been enrolled in the study at its initiation. As a result of this and the subject’s subsequent withdrawal from the study, they were not included in the final study report. Five subjects mentioned reactions or discomfort in the diary (see Table S7). These are incorporated in the results. Three participants developed three adverse events that were not considered to be related to the product, and treatment was not interrupted: a headache, migraine, and dental treatment.

## Discussion

Many rosacea patients report heightened skin sensitivity with skincare and personal hygiene products.^10^ In addition, rosacea patients often experience dry facial skin, which can exacerbate symptoms.^12^ Moisturizers relieve dry skin and improve skin characteristics, such as softness and suppleness, and may improve skin homeostasis and repair defective barrier function.^12^ In addition, skincare products for people with rosacea should block UV light, a leading rosacea trigger.^3^,^11^

Against this background, this open-label, single-center investigation assessed 21 days’ treatment with Daylong Extreme SPF 50+ lotion, a novel SPF 50 moisturizer, in healthy adults with rosacea-prone sensitive skin. The dermatologists’ assessment showed that applying the product once daily for 21 days significantly improved erythema, dryness, and scaling. The formulation was water-based and contained five UV filters, sun protection being critical in rosacea treatments. The product also contained two emollients and three ingredients that condition the skin, accounting for the reduction in dryness and improvement in skin condition. The reduction in facial erythema is consistent with an improvement in stratum corneum-barrier function, which would reduce the ability of irritants to penetrate the skin. Improvements in dryness and scaling is consistent with increased stratum corneum hydration.^12^

Subjects’ assessments of significant improvements in tension and the feeling of dryness concurred with the dermatologists’ findings. This suggests that enhanced stratum corneum-barrier function and hydration produces a symptomatic improvement. Despite subjects with pronounced skin sensitivity being enrolled, the dermatologists’ and subjects’ assessments showed that the product exhibited very good skin tolerability. Only one subject withdrew during the study, and association with the product was considered to be possibly related.

Participants found that the product was easy to apply, and most had a positive overall impression. The product was cosmetically acceptable, and 80% of participants would buy it and recommend it to family and friends. Half would switch to the product. A product that is well accepted by subjects is likely to help ensure good adherence, especially during long-term use, although further studies with longer follow-up are needed to evaluate this. The study was conducted during the summer months, which has clear implications for skin condition. Future studies could assess seasonal variation in treatment outcomes. A more balanced sex ratio would also be of benefit in future studies. Results from the subject evaluation suggest that the product protects against UV exposure, improves redness, dryness, and tightness, and can be included easily in the skincare routine of subjects with sensitive rosacea-prone skin.

## Conclusion

Daylong Extreme SPF 50+ lotion was very well tolerated on the faces of rosacea-prone individuals, producing objective and subjective improvements in skin status and symptoms. The short-term acceptability of the product was rated very highly, in particular with regard to the easy application of the product. The long-term acceptability also yielded positive results, with a significant majority of subjects indicating a positive overall impression of the product.

Agreement was significantly higher regarding positive statements on the product, including its easy application, its moisturizing effects, and prevention of sun-provoked facial redness, as well as its protection from the sun. In addition, a significant majority of the subjects had positive reviews on the texture, color, and scent of the product.

In summary, the test product Daylong Extreme SPF 50+ lotion was very well tolerated on the faces of 98% of subjects with rosacea-prone skin. Considering the skin sensitivity of the subjects involved in the study, the tolerability of the product can be considered extremely favorable, with positive short- and long-term acceptability. Most participants would purchase and recommend the product.

## Figures and Tables

**Table 1:** Dermatological evaluation of skin status at baseline (day 0) and day 22

	Mean values		Median values		Day 0 ≠ day 22	
	Day 0	Day 22	Day 0	Day 22	n (n lower, day 22)	*P*-value
**Erythema**	1	0.4	1	0	34 (32)	<0.001*
**Dryness**	0.2	0	0	0	13 (12)	0.003*
**Scaling**	0.2	0	0	0	12 (11)	0.005*
**Fissures**	0	0	0	0	0	1
**Papules**	0	0.1	0	0	3 (1)	0.75
**Pustules**	0.1	0	0	0	4 (3)	0.75
**Edema**	0	0	0	0	0	1
**Vesicles**	0	0	0	0	0	1
**Weeping**	0	0	0	0	0	1
**Other**	0	0	0	0	0	1

**Notes:**

* *P*<0.05. Evaluation was based on a 5-point scale: 0, no reaction; 0.5, very slight; 1, slight; 2, moderate; 3, strong.

**Table 2:** Subject evaluation of skin status at baseline (day 0) and day 22

	Mean values		Median values		Day 0 ≠ day 22	
	Day 0	Day 22	Day 0	Day 22	n (n lower, day 22)	*P*-value
**Itching**	0.1	0	0	0	2 (2)	0.5
**Burning**	0	0.1	0	0	5 (2)	1
**Tension**	0.7	0.4	0.5	0	23 (18)	0.015*
**Tickling**	0.2	0.1	0	0	13 (9)	0.429
**Dryness**	1	0.5	1	0	30 (24)	0.003*
**Other**	0	0	0	0	1 (0)	1

**Notes:**

* *P*<0.05. Evaluation was based on a 5-point scale: 0, no reaction; 0.5, very slight; 1, slight; 2, moderate; 3, strong.
